# Is there a relationship between screening for diabetes related foot complications and limb amputation?

**DOI:** 10.1186/1757-1146-8-S2-O11

**Published:** 2015-09-22

**Authors:** Ainslie Davies, Peter Lazzarini, Bishnu Sharma, Lloyd Reed

**Affiliations:** 1 School of Clinical Sciences, Queensland University of Technology, Brisbane, Qld, 4590, Australia; 2 Institute of Health and Biomedical Innovation, Queensland University of Technology, Brisbane, Queensland, Australia; 3 Allied Health Research Collaborative, Metro North Hospital & Health Service, Brisbane, Queensland, Australia; 4 School of Business, University of the Sunshine Coast, Qld, 4556, Australia

**Keywords:** Diabetic foot screening, amputation, primary care

## Background

From the conservative estimates of registrants with the National Diabetes Supply Scheme, we will be soon passing 1.1 Million Australians affected by all types of diabetes. The diabetes complications of foot ulceration and amputation are costly to all. These costs can be reduced with appropriate prevention strategies, starting with identifying people at risk through primary care diabetic foot screening. Yet levels of diabetic foot screening in Australia are difficult to quantify. This presentation aims to report on foot screening rates as recorded in existing academic literature, national health surveys and national database reports.

## Methods

Literature searches included diabetic foot screening that occurred in the primary care setting for populations over 2000 people from 2002 to 2014. Searches were performed using Medline and CINAHL as well as internet searches of Organisations for Economic Co-operation and Development (OECD) countries health databases. The focus is on type 1 and type 2 diabetes in adults, and not gestational diabetes or children. The two primary outcome measures were foot -screening rates as a percentage of adult diabetic population and major lower limb amputation incidence rates from standardised OECD data.

## Results

The most recent and accurate level for Australian population review was in the AUSDIAB (Australian Diabetes and lifestyle survey) from 2004. This survey reported screening in primary care to be as low as 50%. Countries such as the United Kingdom and United States of America have much higher reported rates of foot screening (67-86%) recorded using national databases and web based initiatives that involve patients and clinicians. By comparison major amputation rates for Australia were similar to the United Kingdom at 6.5 versus 5.1 per 100,000 population, but dis-similar to the United States of America at 17 per 100,000 population.

## Conclusions

Australian rates of diabetic foot screening in primary care centres is ambiguous. There is no direct relationship between foot screening levels in a primary care environment and major lower limb amputation, based on national health survey's and OECD data. Uptake of national registers, incentives and web-based systems improve levels of diabetic foot assessment, which are the first steps to a healthier diabetic population.

